# Phytochemical Profile and Assessment of In Vivo Anti-Inflammatory Efficacy of *Ficus sycomorus* L. (Moraceae) Extracts in Mice

**DOI:** 10.1155/sci5/8849948

**Published:** 2025-09-23

**Authors:** Stephen Maina Gitahi, Eunice Wothaya Muthee, Mathew Piero Ngugi, Alex Kingori Machocho

**Affiliations:** ^1^Department of Natural Sciences, Catholic University of Eastern Africa, P.O. Box 62157-00200, Nairobi, Kenya; ^2^Department of Biochemistry, Microbiology, and Biotechnology, Kenyatta University, P.O. Box 43844-00100, Nairobi, Kenya; ^3^Department of Chemistry, Kenyatta University, P.O. Box 43844-00100, Nairobi, Kenya

**Keywords:** anti-inflammation, edema, *F. sycomorus*, leaf, phytocompounds, stem bark

## Abstract

Inflammation helps the immune system identify and eliminate disease-causing and foreign stimuli and initiate the healing process. Nonsteroidal anti-inflammatory drugs have been often used in treating inflammation. Synthetic drugs have been associated with severe effects, necessitating the need for alternative medicinal agents. Herbal remedies have comparatively fewer side effects, are widely available, and are arguably affordable, which makes them more attractive therapeutic agents. *Ficus sycomorus* is utilized by Kenya's *Mbeere* community to treat inflammation. However, the science-based data to support their claim were lacking. The purpose of this study was to ascertain whether methanol (MeOH) and dichloromethane (DCM) leaf and stem bark extracts of *F*. *sycomorus* have anti-inflammatory qualities in mice. Gas chromatography–mass spectrometry (GC–MS) andliquid chromatography–mass spectrometry (LC–MS) were used in phytochemical analysis of the extracts. In anti-inflammatory assays, 6–7-week-old mice were randomly grouped into six clusters with five mice each. Group one mice were administered normal saline. Groups of two to four mice were injected with carrageenan to induce inflammation and then received various treatments. Group two mice received the vehicle (normal saline), while group three mice received diclofenac (15 mg/kg body weight [bw]). The extracts were administered to the remaining groups at 50, 100, and 200 mg/kg bw. One-way analysis of variance was used to assess for statistically significant differences, followed by Tukey's post hoc tests in case of statistical significance. The significance threshold was inferred at *p* < 0.05. This study revealed significant anti-edema effects of the extracts on carrageenan-induced paw inflammation in mice. The GC–MS analysis identified fatty acids, terpenoids, and terpenes, which have been associated with anti-edema effects. In conclusion, the findings showed that the extracts have anti-edema effects and phytocompounds associated with the effects. *F. sycomorus* extract is hence a novel candidate for developing efficacious anti-inflammatory agents.

## 1. Introduction

The term “inflammation” (phlogosis) is defined as the body's immunological reaction to tissue damage caused by exposure to infections, radiation, and noxious chemicals, as well as trauma. It is characterized by pathophysiological responses such as accumulation of blood cells and plasmatic fluid in the site of injury and helps the body's immune system to identify and fight pathogens and facilitate the healing process [1]. Acute and chronic inflammations are the two known types of inflammation. The former is a short-term reaction with a localized effect and responds where a problem exists, whereas chronic inflammation has a long-term and whole-body effect and produces mild inflammation in the entire body, which contributes to the progression of illnesses such as psoriasis, colitis, multiple sclerosis, rheumatoid arthritis, and chronic asthma, bronchitis, and dermatitis [2].

Globally, the leading causes of significant mortality are chronic inflammatory diseases. The World Health Organization (WHO) recognizes chronic illnesses as a public health concern. Over 50% of all deaths worldwide are attributable to chronic inflammatory diseases, including neurodegenerative ailments, chronic kidney disease, autoimmune disorders, stroke, liver disease, diabetes mellitus, cancer, and ischemic heart disease [3].

Although inflammation is important to the well-being of the body, it subjects individuals to discomfort and distress. Essential inflammation may be irritating to healthy tissues, whereas excessive inflammatory reaction presents deleterious effects on an individual [4]. Nonsteroidal anti-inflammatory drugs like ibuprofen, etoricoxib, celecoxib, naproxen, indomethacin, and diclofenac are frequently used to treat inflammation. Nonetheless, these conventional medicines are known to possess numerous side effects, including gastrointestinal, kidney, liver, and heart toxicities, among others [3, 5, 6].

The WHO estimates that 80 percent of people in underdeveloped nations treat illnesses and disorders using medicinal plants [3, 7, 8].

Recently, botanical-based products (nutritional, agricultural, and medicinal) have increased commercial significance in a myriad of pharmaceutical, agro-based, and nutraceutical industries [9].

The demand for herbal medicines has risen as a result of consumers realizing that natural products have fewer adverse effects, are accessible, and are more affordable [10]. Because of their cultural beliefs, many people in rural areas use herbal treatments on a sporadic basis [11]. The usage of herbal remedies has grown significantly in popularity and worldwide during the past 10 years. As the use of herbal medicines spreads throughout the world, quality monitoring of these products is becoming the public's and health authorities' top concern [12].

Herbal formulations or natural products are crucial sources in the development of novel anti-inflammatory therapeutics. Under stressful conditions, medicinal plants often synthesize secondary metabolites. These metabolites possess various pharmacological effects against various diseases and disorders, including inflammation [13]. The value of medicinal plants depends on various bioactive compounds such as saponins, flavonoids, tannins, alkaloids, steroids, phenolic acids, and terpenoids. These phytocompounds are associated with anti-inflammatory effects [13, 14].

Currently, studies are being carried out to establish whether traditional use of medicinal plants is supported by empirical scientific evidence. *Ficus sycomorus* L., commonly known as mulberry fig, has been used in the treatment of inflammation among communities in Embu County in Kenya. However, there is a paucity of scientific evidence to confirm their acclaimed use. The current investigation, therefore, aimed to determine the anti-inflammatory and quantitative phytochemical composition of dichloromethane (DCM) and methanolic extracts of *F. sycomorus* L.

## 2. Materials and Methods

### 2.1. Medicinal Plant Sample Collection

Based on ethnobotanical knowledge obtained from a local traditional herbalist in Embu County, Kenya, fresh leaves of *F. sycomorus* were selected and collected. Plant materials were gathered using appropriate bio-conservation techniques. Using a handheld Global Positioning System device (model type Garmin Etrex H), the coordinates where the medicinal plant samples were collected were obtained and recorded as “South West 68° 0 36′ 33″ S, 37° 3 7′ 15″ E.” They were later transported to the National Museums of Kenya in the Department of Botany, herbarium section, where botanical authentication was done by an acknowledged taxonomist. After being given a voucher number (EWM-001), the plant sample was saved in the National Museums of Kenya herbarium for further use.

### 2.2. Preparation of Plant Materials

The medicinal samples underwent a thorough sorting, cleaning, cutting into small pieces, and 2 weeks of shade drying. The dried plant materials were ground using an electric mill and stored in paper bags ready for extraction.

### 2.3. Extraction

*F. sycomorus* powdered leaves weighed three hundred grams (300 g) and were placed in a conical flask and filled with 1 L of DCM or methanol (MeOH). The mixtures were left to stand for 24 h, with frequent spinning every 6 h. Filtration was carried out, followed by concentration using a rotary evaporator. The DCM and MeOH filtrates were concentrated at 64°C and 45°C, respectively, and under decreased pressure to produce a semisolid extract. The semisolid extracts were allowed to dry, weighed using a precision weighing balance, and then refrigerated at 4°C awaiting phytochemical analysis and bioassays. A formula used by Moriasi et al. [15] was used to calculate percentage extract yields:

(1) $$\begin{array}{c} \%\text{ Extract Yield}=\frac{\text{Mass of the obtained extract }}{\text{Mass of the powdered sample}}\times 100. \end{array}$$

### 2.4. Evaluation of In Vivo Anti-Inflammatory Effects

#### 2.4.1. Experimental Animals

Swiss albino mice, *Mus musculus* (female), weighing 20–22 g, and aged 7–8 weeks. The mice were purchased and raised in the Animal Breeding and Research Facility at Kenyatta University. The mice were acclimatized for 48 h before experimentation. They were maintained in standard propylene cages under ambient room temperature 25°C with 12 h of daylight. The animals were supplied with water *ad libitum* and nourished with the standard mice pellet (Unga Holding Limited, Kenya). The animals were handled ethically, minimizing any discomfort to the animals. The Kenyatta University's Ethics Committee for the Care and Use of Laboratory Animals granted ethical approval for the use of research animals (Approval number PKUA/007/007).

#### 2.4.2. Induction of Inflammation

Inflammation was induced in mice through injection of carrageenan (0.1 mL, 1% commercial grade type 1) solution into left hind paw subplantar tissue. Inflammation was confirmed by measuring paw diameter before and after induction using digital vernier calipers.

### 2.5. Study and Experimental Design

This investigation used a completely random controlled experimental study design. An experimental design was derived from the study design. Randomly, mice were assigned to 6 groups (*n* = 5): negative control (edema control), normal control (non-edema control), diclofenac control (positive control), and 3 dosage extract-treated mice. The normal control mice received normal saline (vehicle) containing 5% DMSO. After an hour of edema induction, the edema control mice were administered the vehicle. An hour after the diclofenac control mice were induced with inflammation, they were administered diclofenac sodium (15 mg/kg bw [body weight]). The extract-treated mice were induced with edema 1 h before administration of the *F. sycomorus* extracts at 50, 100, and 200 mg/kg bw. The anti-inflammatory assay was conducted following the protocol as described by Umamageswari and Yasmeen [16] (Table 1).

The paw diameter was measured 30 min before injecting carrageenan and then in the 1st, 2^nd^, 3^rd^, and 4^th^ hours after treatment. The formula by Umamageswari and Yasmeen [16] was utilized to compute the percent (%) inhibition of edema.

(2) $$\begin{array}{c} \text{Percentage Inhibition}=\frac{V_{c}-V_{t}}{V_{t}}\times 100, \end{array}$$

where *V*_*c*_ = edema volume in the control group and *V*_*t*_ = edema diameter in the treated group.

### 2.6. Phytochemical Screening

#### 2.6.1. Gas Chromatography–Mass Spectrometry (GC–MS)

One gram of DCM leaf and stem bark extract of *F. sycomorus* were each dissolved in 1 mL of DCM (Sigma-Aldrich grade). A 1.5-mL Eppendorf tube was used to weigh the samples, and each weight was recorded in milligrams. Each sample was dissolved in one mL of DCM, vortexed for 30 s, sonicated for 15 min in an ultra-bath, and then centrifuged at 10,000 rpm for 5 min. Subsequently, the samples were placed in 2 mL autosampler vials and then subjected to GC–MS analysis. Each extract was injected at a volume of 1 μL.

The plant extracts were analyzed using the following parameters using an Agilent Gas Chromatograph (7683 Agilent Technology, Inc., Beijing, China) connected to a 5975c inert XL EI/CI Mass Spectrometer in full scan mode gas chromatography. Column HP_5MS is a low-bleed capillary with a 0.25 mm diameter, 0.25 μm film thickness, and 30 m length that contains 5% phenyl methyl siloxane. Before analyzing the extracts, the temperature of the oven, the flow rate of helium, and the electron gun of the gas chromatography and mass spectrometry were programmed. An electron ionization apparatus with an ionization energy of 70 eV was used during analysis.

At a steady flow rate of 7.2 mL per minute, helium (99.99%) was utilized as the carrier gas. The injector and mass transfer line temperatures were adjusted to 250°C and 200°C, respectively. The oven was programmed to operate at 50°C/minute for 50 min, reaching 285°C in 9 min, with a 50 min run time. The oven's original operating temperature was 35°C for five minutes, followed by a 10-degree Celsius rise every minute to 280°C for 10 minutes. The mass spectrometry was operated with the following parameters: 250°C for the interface temperature, 1666 µsec for scan speed, 40–550 M/Z for scan range, 70 eV for ionization energy, and 50–70 min for total retention period.

The concentration of each component in the sample was calculated using the beta-sitosterol linear equation *y* = 186096*x* + 1000000. The spectrum of the known components kept in the National Institute of Standard and Technology computer library was used to identify unknown phytocompounds.

#### 2.6.2. Liquid Chromatography–Mass Spectrometry (LC–MS)

Samples (1 g) were each dissolved in 1 mL (90:10 MeOH, deionized distilled H_2_O), vortexed for 10 s, sonicated for 30 min, centrifuged at 14,000 rpm for 10 min at 4°C, and then the supernatant (0.1 μL) analyzed utilizing ultra-performance liquid chromatography–mass spectrometry (UPLC–MS). The ACQUITY UPLC I-Class apparatus from Waters Corporation in Milford, Massachusetts, was utilized to perform the chromatographic separation. It was outfitted with an ACQUITY UPLC BEH C18 column (Waters Corporation, Wexford, Ireland) (2.1 × 150 mm, 1.7 μm particle size; oven temperature 45°C). A steady flow rate of 0.2 mL per minute was maintained.

Two solvents, water and MeOH, each acidified with formic acid (0.01%), formed the mobile phase. The gradient system that was utilized was 0–2 min, 5% B; 2–4 min, 40% B; 4–7 min, 40% B; 7–8.5 min, 60% B; 8.5–10 min, 60% B; 10–15 min, 80% B; 15–19 min, 80% B; 19–20.5 min, 100% B; 20.5–23 min, 100% B; 23–24 min, 95% B; and 24–26 min, 95% B. The autosampler tray was cooled to 5°C.

The ultra-performance liquid chromatography was interfaced with an electrospray ionization (ESI) in positive ionization mode. The settings that were used were the mass-to-charge ratio (m/z) range of 40–2,000, the sampling cone voltage of 30 V, the capillary voltage of 0.5 kV, the source temperature of 150°C, and the desolvation temperature of 120°C. The flow rate for nitrogen desolvation was 800 L/h.

MassLynx Version 4.1 SCN 712 software was utilized to collect the data. Adducts, common segments, literature, online databases (METLIN, ChemSpider), and, when accessible, co-injections to validate with genuine samples were used to generate molecular ion peaks. After the mass spectra for each peak were generated, possible phytochemicals were established. The linear equation of the oleic acid standard (*y* = 6181.1*x* + 21,034) was used to calculate the concentration of the components.

### 2.7. Statistical Data Analysis

The data were cleaned and then organized for analysis utilizing Minitab statistical software Version 21.0. Means ± SEM (standard error of the mean) were computed to generate descriptive statistics. To ascertain the statistical variations between the various test groups, the inferential statistic one-factorial ANOVA (analysis of variance) was employed. In case of statistical variations, pairwise mean comparisons were performed using Tukey's multiple comparisons. An independent/unpaired *t*-test was employed to compare the effects of the two extracts. A *p* < 0.05 was used to define a significance level. Tables and figures were employed to illustrate the analyzed data.

## 3. Results

### 3.1. In Vivo Anti-Inflammatory Activities of DCM and MeOH Stem Bark Extracts of *F. sycomorus*

The administration of DCM stem bark extract reduced the inflamed paw diameter of mice in the treatment period, as indicated in Table 2. However, the percentage change in paw edema in the edema control mice was substantially higher relative to mice that received diclofenac and DCM stem bark extract at 50, 100, and 200 mg/kg bw from the 1^st^ of treatment onward (*p* < 0.05). The extract showed a response that was dosage dependent in the 1^st^, 3^rd^, and 4^th^ hours (Table 2).

The inflamed paw diameter was reduced by 4.02%, 6.51%, and 6.68% in the 1^st^ hour by the *F*. *sycomorus* DCM stem bark extract at 50, 100, and 200 mg/kg bw, respectively. The anti-edema effects of DCM stem bark extract varied significantly between the three doses (*p* < 0.05). Conversely, the anti-edema effect of DCM extract did not vary significantly (*p* > 0.05) at 100 and 200 mg/kg bw in mice. Similarly, the effect of diclofenac (positive control) did not differ significantly compared to the effect of the extract at 50 mg/kg bw (*p* > 0.05) as depicted in Table 2.

The paw diameter was decreased to 92.41%, 91.09%, and 91.31% in the 2^nd^ hour by the *F*. *sycomorus* DCM stem bark extract at 50, 100, and 200 mg/kg bw, respectively. At all three doses, the extract's anti-edema was statistically equivalent to that of diclofenac (*p* > 0.05). The anti-edema effect of the DCM extract was dose-independent, as shown in Table 2.

The DCM extract at 50, 100, and 200 mg/kg bw in the 3^rd^ hour attenuated the paw edema by 10.36%, 11.74%, and 12.2%, respectively. The anti-edema activity of 100 and 200 mg/kg bw DCM extract dosages in mice did not differ significantly (*p* >  0.05). Similarly, the anti-edema effect of diclofenac did not vary considerably compared to the effect of the extract dose of 50 mg/kg bw in mice (*p* > 0.05; Table 2)

The inflamed paw diameter declined to 87.52%, 86.31%, and 85.53% in the 4^th^ hour when *F*. *sycomorus* DCM stem bark extract was administered at 50, 100, and 200 mg/kg bw, respectively. The anti-edema effect of the DCM extract at the three studied doses differed substantially in mice (*p* < 0.05). Nevertheless, the effect of diclofenac did not differ substantially from that of the extract (*p* > 0.05) at the three doses, as illustrated in Table 2.

The MeOH stem bark extract of *F*. *sycomorus* also noted an anti-edema effect following induction of paw edema using carrageenan, as depicted in Table 3. This was noted by the decline in paw diameter after the animals received the extract at 50, 100, and 200 mg/kg bw. However, from the 1^st^ hour onward, the percentage change in paw diameter of edema control mice was significantly higher in contrast to those of diclofenac-treated and extract-treated mice (*p* < 0.05) at the three tested doses. From the first hour onward, the MeOH extracts ameliorated paw edema dose-dependently (Table 3).

The paw inflammation was reduced to 94.88%, 94.07%, 90.33%, and 94% in the 1^st^ hour by using MeOH stem bark extract at 50, 100, and 200 mg/kg bw and diclofenac, respectively. The anti-edema effect of the MeOH extract dosages of 50 and 100 mg/kg bw and diclofenac did not differ significantly in mice, as shown in Table 3 (*p* > 0.05).

The paw inflammation was ameliorated by 8.46%, 10.80%, and 12.68% in the 2^nd^ hour by the extract at 50, 100, and 200 mg/kg bw, respectively. There was no considerable variation in the extract's anti-edema activity across the three doses (*p* < 0.05) in this hour. At 50 mg/kg bw, the anti-edema effect of the extract did not differ significantly from the effect of the diclofenac (*p* > 0.05), as illustrated in Table 3.

The paw inflammation was lowered to 88.99%, 87.08%, 85.17%, and 88.44% in the 3^rd^ hour by MeOH extract at 50, 100, and 200 mg/kg bw, including diclofenac, respectively. The three extract doses showed significantly different anti-edema effects in mice (*p* < 0.05). Nonetheless, the anti-edema effect of the MeOH extract at 50 mg/kg bw did not vary considerably from the effect of diclofenac, as depicted in Table 3 (*p* > 0.05).

In the 4^th^ hour, the mice that received MeOH extract at 50, 100, and 200 mg/kg bw noted a decline in paw inflammation by 12.79%, 14.61%, and 16.54%, respectively. The anti-edema effect of the MeOH extract at the three doses differed substantially (*p* < 0.05). The anti-edema activity of diclofenac statistically matched the extract's activity (*p* > 0.05) at 50 mg/kg bw, as shown in Table 3.

**Figure 1:**
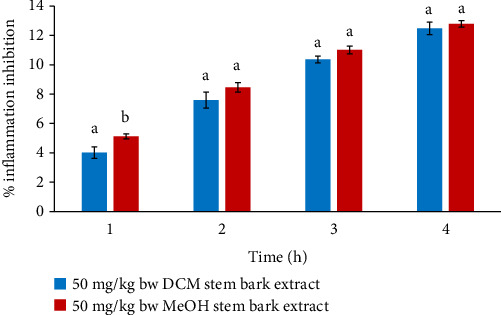
Comparison of in vivo anti-inflammatory activities of DCM (blue) and MeOH (red) stem bark extracts of *F*. *sycomorus* at 50 mg/kg bw. Using an independent *t*-test, bars with similar lowercase letters within the same hour do not differ significantly (*p* > 0.05).

In comparison, the anti-edema effects of DCM and MeOH stem bark extracts at 50 mg/kg bw did not vary in the 2^nd^, 3^rd^, and 4^th^ hours, as illustrated in Figure 1 (*p* > 0.05). Conversely, the paw diameters of those that received MeOH stem bark extract were substantially higher relative to the paw diameter of mice that received DCM stem bark extract (*p* < 0.05) in the 1^st^ hour (Figure 1).

**Figure 2:**
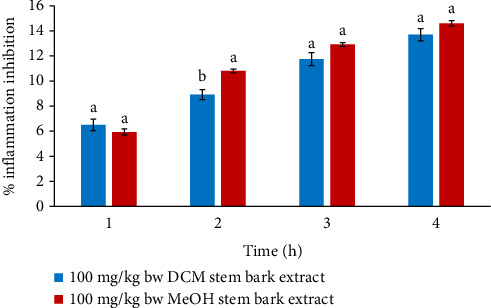
Comparison of in vivo anti-inflammatory activities of DCM and MEOH stem bark extracts of *F*. *sycomorus* at 100 mg/kg bw. Using an independent *t*-test, bars with similar lowercase letters within the same hour do not differ significantly (*p* > 0.05).

At 100 mg/kg bw, the paw diameters of mice that were administered DCM stem bark extract were substantially lower relative to those of mice that received MeOH stem bark in the 2^nd^ hour (*p* < 0.05). Nevertheless, the anti-edema effects of the two extracts were insignificant in the 1^st^, 3^rd^, and 4^th^ hours of the study (*p* > 0.05), as shown in Figure 2.

**Figure 3:**
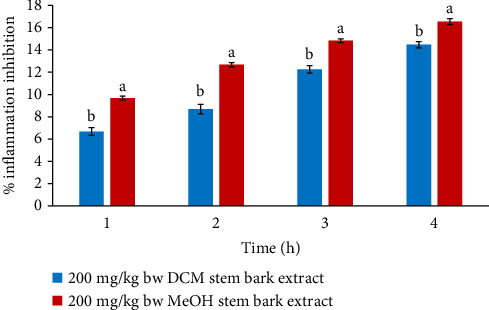
Comparison of in vivo anti-inflammatory activities of DCM and MeOH stem bark extracts of *F*. *sycomorus* at 200 mg/kg bw. Using an independent *t*-test, bars with similar lowercase letters within the same hour do not differ significantly (*p* > 0.05).

At 200 mg/kg bw, the paw diameters of mice treated with DCM extract were considerably lower relative to the paw diameter of MeOH extract in the entire treatment period (*p* < 0.05; Figure 3).

### 3.2. In Vivo Anti-Inflammatory Activities of DCM and MeOH Leaf Extracts of *F. sycomorus*

In general, the DCM leaf extract of *F. sycomorus* at 50, 100, and 200 mg/kg bw noted an anti-edema effect in mice that were induced with inflammation, as illustrated in Table 4. This effect was revealed by a decline in paw inflammation following therapy with the extract. The percentage change in paw diameter in edema control mice was substantially greater relative to the paw diameter of mice administered with diclofenac and leaf extract at all dose levels from hour 2 onward (*p* < 0.05). In the entire treatment period, the anti-edema effect of the extract reduced the paw edema dose-dependently (Table 4).

The DCM extract at 50, 100, and 200 mg/kg bw and diclofenac ameliorated the inflamed paw by 4.50%, 4.37%, 4.72%, and 5.29% in the first hour, respectively. The anti-edema effect of the DCM extract at 50, 100, and 200 mg/kg bw did not vary considerably relative to the activity (*p* > 0.05) of diclofenac, as shown in Table 4.

The DCM extract at 50, 100, and 200 mg/kg bw and diclofenac alleviated the diameter of the inflamed paw by 7.63%, 8.15%, 8.19%, and 8.24%, respectively, in the second hour. The anti-edema activity of DCM extract at 50, 100, and 200 mg/kg bw did not differ considerably and was comparable to the effect of diclofenac (*p* > 0.05), as shown in Table 4.

The leaf extract at 50, 100, and 200 mg/kg bw ameliorated the inflamed paw by 9.20%, 9.93%, and 11.48% in the third hour, respectively. The extract's anti-edema effect at 50 and 100 mg/kg bw did not differ substantially in mice (*p* > 0.05). The anti-edema effect of diclofenac did not differ statistically compared to the effect of the extract at 200 mg/kg bw (*p* > 0.05) as detailed in Table 4.

The inflamed paw diameter was reduced by 10.95%, 12.29%, 13.73%, and 12.91% in the fourth hour by *F*. *sycomorus* DCM leaf extract at 50, 100, and 200 mg/kg bw and diclofenac, respectively. The anti-inflammatory effect of DCM extract at 50, 100, and 200 mg/kg bw differed considerably in mice (*p* < 0.05). The effect of diclofenac did not vary considerably compared to the effect of the DCM extract at 100 and 200 mg/kg bw (*p* > 0.05) as detailed in Table 4.

Moreover, the MeOH leaf extract of *F*. *sycomorus* at 50, 100, and 200 mg/kg bw ameliorated paw edema. The percentage change in paw diameter of edema control mice was a considerably higher relative percentage change than that of mice (*p* < 0.05) in the other studied groups. In the entire treatment period, the extract reduced paw edema in a dose-dependent manner (Table 5).

The inflamed paw diameter was reduced by 2.67%, 3.42%, 3.94%, and 3.91% in the 1^st^ hour by the *F*. *sycomorus* MeOH leaf extract at 50, 100, and 200 mg/kg bw and by diclofenac, respectively. The anti-inflammatory effect of the MeOH extract at 50, 100, and 200 mg/kg bw did not vary substantially and statistically matched the effect of diclofenac (*p* > 0.05) as depicted in Table 5.

The MeOH extract at 50, 100, and 200 mg/kg bw in the 2^nd^ hour alleviated paw edema to 94.48%, 93.77%, and 93.11%, respectively. The anti-inflammatory activity of the extract at 50 and 100 mg/kg bw did not vary considerably in mice (*p* > 0.05). Additionally, the anti-edema effect at 100 and 200 mg/kg bw of the extract also noted statistical similarities (*p* > 0.05). Further, the effect of diclofenac statistically matched that of leaf MeOH extract at 200 mg/kg bw in mice (*p* > 0.05) as presented in Table 5.

The paw edema was reduced by 8.75%, 10.45%, 11.83%, and 10.70% in the 3^rd^ hour by MeOH leaf extract at 50, 100, and 200 mg/kg bw and diclofenac, respectively. The anti-edema effect of the extract at 100 and 200 mg/kg bw did not vary considerably and was comparable (*p* > 0.05)to that of diclofenac (Table 5).

The paw diameter was reduced to 88.97%, 87.93%, 85.23%, and 86.86% in the 4^th^ hour by MeOH leaf extract at 50, 100, and 200 mg/kg bw and diclofenac, respectively. The anti-edema effect of the extract at three studied doses varied significantly in mice (*p* < 0.05). The effect of diclofenac was substantially lower relative to the effect of the extract at 200 mg/kg bw in mice (*p* < 0.05), as illustrated in Table 5.

**Figure 4:**
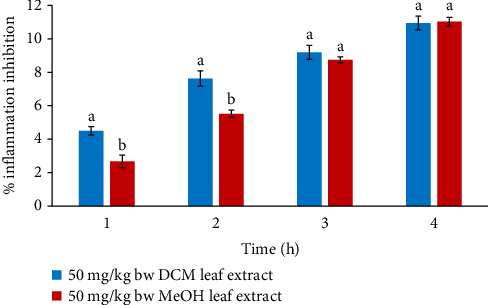
Comparison of in vivo anti-inflammatory effects of DCM and MeOH leaf extracts of *F. sycomorus* at 50 mg/kg bw. Using an independent *t*-test, bars with similar lowercase letters within the same hour do not differ significantly (*p* > 0.05).

In comparison, the anti-edema effect of MeOH leaf extract at 50 mg/kg bw significantly reduced paw edema compared to the effect of DCM extract at the corresponding dosage (*p* < 0.05) in the 1^st^ and 2^nd^ hours. Nonetheless, there was no significant difference in the anti-edema effects of DCM and MeOH leaf extracts (*p* > 0.05) at 50 mg/kg bw in mice in the 3^rd^ and 4^th^ hours (Figure 4).

**Figure 5:**
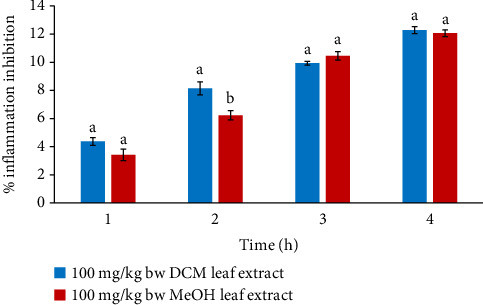
Comparison of in vivo anti-inflammatory effects of DCM and MeOH leaf extracts of *F*. *sycomorus* at 100 mg/kg bw. Using an independent *t*-test, bars with similar lowercase letters within the same hour do not differ significantly (*p* > 0.05).

The anti-edema effects of MeOH and DCM leaf extracts at 100 mg/kg bw did not vary considerably in the 1^st^, 3^rd^, and 4^th^ hours of the study (*p* > 0.05). Nonetheless, the anti-edema effect of the DCM extract dose of 100 mg/kg bw was considerably greater relative to that of MeOH leaf extract at the same dose in the second hour (*p* < 0.05), as illustrated in Figure 5.

**Figure 6:**
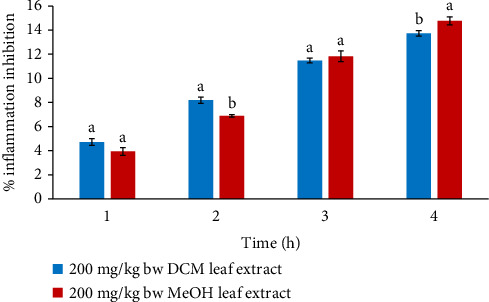
Comparison of in vivo anti-inflammatory effects of DCM and MeOH leaf extracts of *F. sycomorus* at 200 mg/kg bw. Using an independent *t*-test, bars with similar lowercase letters within the same hour do not differ significantly (*p* > 0.05).

In the first and third hours, the anti-edema effects of the two leaf extracts at 200 mg/kg bw were insignificant in mice (*p* > 0.05). Nevertheless, the anti-edema of the DCM leaf extract at 200 mg/kg bw varied significantly in contrast to that of the MeOH leaf extract (*p* < 0.05) in the 2^nd^ and 4^th^ hours, as depicted in Figure 6.

### 3.3. Phytochemical Compositions

#### 3.3.1. LC–MS Analysis of MeOH Extracts of *F. sycomorus*

**Figure 7:**
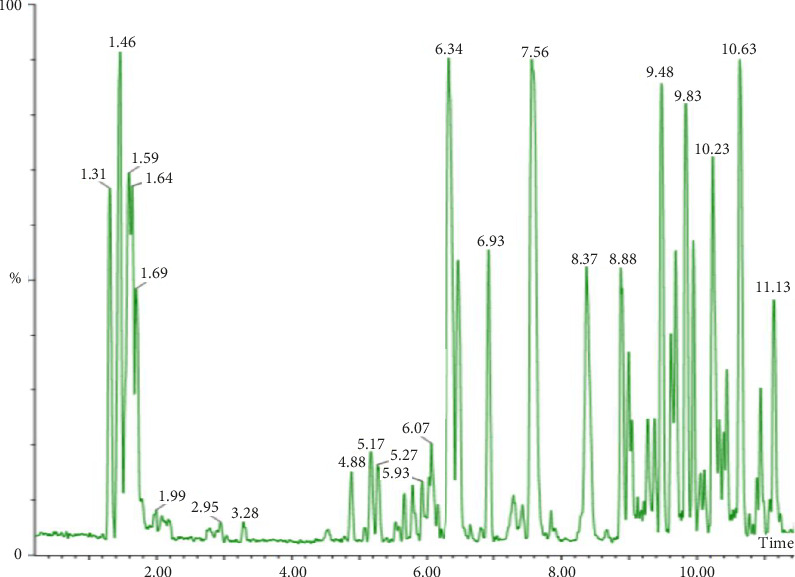
Chromatogram of LC–MS analysis of MeOH stem bark extract of *F*. *sycomorus*.

The LC–MS analysis of MeOH stem bark extract of *F*. *sycomorus* detected 20 phytochemical compounds belonging to 10 classes (Table 6; Figure 7). These included tannin (1), phenolics (2), phenols (2), flavonoids (6), fatty acids (2), flavonols (2), ester (1), sterols (2), phytosterol (1), and isoflavone (1). Ferulic had the most abundance of 16.87%, followed by catechin with an abundance at 14.02%. The least percentage abundance of 0.09% was reported in stigma sterol, as shown in Table 6.

**Figure 8:**
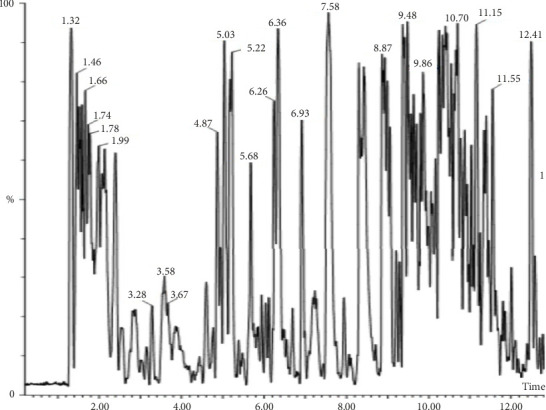
Chromatogram of LC–MS analysis of *F*. *sycomorus* MeOH leaf extract.

The LC–MS analysis of MeOH leaf extract of *F. sycomorus* detected 18 phytochemical compounds belonging to 7 classes (Table 7; Figure 8). These included phenolics (3), flavonoids (7), fatty acids (2), flavonols (2), ester (1), sterols (2), and isoflavone (1). Ferulic acid had the highest abundance of 14.27%, followed by genistein with an abundance of 10.69%. The least percentage abundance of 1.86% was reported in procyanidin B2 (Table 7).

#### 3.3.2. GC–MS Analysis of DCM Extracts of *F. Sycomorus*

**Figure 9:**
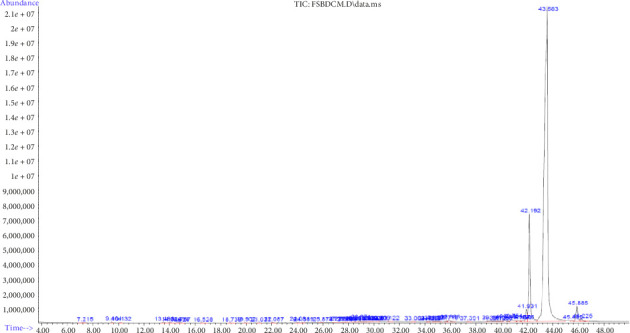
Chromatogram of GC–MS analysis of *F*. *sycomorus* DCM stem bark extract.

The GC–MS analysis of DCM stem bark extract identified 20 phytocompounds with different retention times (Table 8; Figure 9). The phytochemicals were classified into different classes, including alkaloid, alkene, pyrazine, alcohol, cyclic amine, furan, fatty acid methyl ester, sesquiterpenoids, monoterpenoid, fatty acid, and phytosterol, as detailed in Table 4. The most abundant phytocompound was an alcohol, A-neogammacer-22(29)-en-3-ol (82.60%), followed by the sesquiterpene, zierone (14.93%) (Table 8).

**Figure 10:**
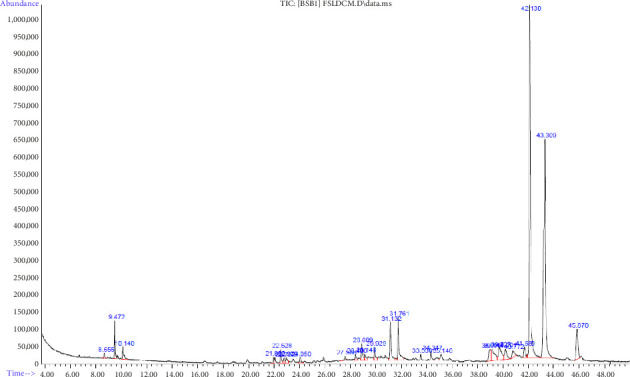
Chromatogram of GC–MS analysis of *F*. *sycomorus* DCM leaf extract.

About 25 phytocompounds with different retention times and belonging to different phytochemical classes were detected using GC–MS analysis (Table 9; Figure 10). The phytocompounds belonged to classes of alkenes, dicarboxylic acid, aldehyde, monoterpene, fatty acid, acyclic diterpenoids, esters, fatty alcohol, carboxylic esters, fatty acid methyl ester, diterpene, vitamin, phytosterol, triterpenes, sesquiterpene, and alcohol, as illustrated in Table 4. The most abundant phytochemical was zierone (sesquiterpene) at 40.68% (Table 9).

## 4. Discussion

Inflammation causes suffering and minimizes victims' productivity [17, 18]. The NSAIDs and corticosteroid drugs are often prescribed to treat inflammation. Nevertheless, these drugs are associated with hepatic, renal, and gastrointestinal toxicities [19, 20]. This necessitates the need for alternative therapeutic agents. Medicinal plants are largely used to manage diseases and disorders, particularly in developing nations [7]. *F. sycomorus* is used by Kenyan communities to manage inflammation and pain, among other uses. However, the scientific data to validate these claims were lacking. This investigation aimed to determine the in vivo anti-edema potential and quantitative phytochemical profiles of DCM and MeOH extracts of *F. sycomorus*.

In the current study, the *F. sycomorus* MeOH and DCM stem bark and leaf extracts revealed anti-edema effects following induction of paw edema using carrageenan in mice. This was demonstrated by an attenuation of paw edema following administration of the extracts at the three doses in mice. Generally, MeOH extracts have more polar, nonvolatile, and high-molecular-weight compounds than DCM, which extracts mostly nonpolar, volatile, and low-molecular-weight compounds [3, 21, 22]. This suggested that both polar and nonpolar compounds had anti-inflammatory potential.

The findings of the current investigation corroborate with that of Alemu et al. [23], who demonstrated the anti-edema effect of *Leonotis ocymifolia* (Burm. F.) MeOH extract following induction of paw edema using carrageenan in mice? Besides, Asefa et al. [24] documented that MeOH extract of *Verbascum sinaiticum* Benth had an anti-edema effect following induction of paw edema using carrageenan in mice. Further, Kiambi Mworia et al. [25] demonstrated that *Senna didymobotrya* (Fresenius) and *Eucalyptus globulus* (Labill) DCM leaf extracts had anti-edema effects following induction of paw edema using carrageenan in mice. Moreover, Veronica et al. [26] found that *Acacia mellifera* DCM stem bark extract had an anti-edema effect following induction of paw edema using carrageenan in mice.

The most widely used standard model for acute inflammatory research to assess the anti-edematous effects of medicinal plants is carrageenan-induced paw inflammation [27]. This model produces reproducible results; hence the choice in this study. Edema caused by carrageenan is classified into two phases. The 1^st^ phase occurs after injection of carrageenan and produces proinflammatory mediators, including bradykinins, histamines, serotonins, and reactive oxygen species [28]. These proinflammatory mediators promote instant onset of edema, hyperalgesia, and erythema [27]. Increased vascular permeability and venous blockage are the primary causes of peripheral edema [29]. The second phase typically appears 3 h after injection of carrageenan. Prostaglandins are the primary players in this phase, including TNF-α, IL-1β, and IL-6 [28, 30].

In this study, the changes in paw edema thickness were utilized to establish the anti-edema potential of the extracts. Carrageenan caused an increased paw edema 1 h after the injection, and then the edema subsided after 4 h. The DCM stem bark extract produces a dose-dependent response in the 1^st^, 3^rd^, and 4^th^ hours of the study. The MeOH and DCM stem bark extracts had dose-dependent responses throughout the experiment. The higher dose (200 mg/kg bw) had better anti-inflammatory effects than the lower dose (50 mg/kg bw). This may be ascribed to rapid metabolism and clearance, as well as deactivation of bioactive substances at lower than higher dosages [26]. It was also noted that the four extracts were more efficacious in the 3^rd^ and 4^th^ hours of the study. This could be ascribed to the gradual passive diffusion of anti-inflammatory components in the peritoneal cavity through the cell membrane [25].

In the current study, diclofenac, a nonsteroidal anti-inflammatory drug, was chosen as the reference drug. Diclofenac exerts an anti-edema effect by blocking COX-2 activity. This inhibits the synthesis of PGE2, one of the essential components of inflammatory mediators [31]. The four extracts in this study are believed to have suppressed the synthesis of PGE2, including other inflammatory mediators such as histamines, bradykinins, serotonins, proinflammatory cytokines, and reactive oxygen species. This could be attributed to phytocompounds that were identified using LC–MS and GC–MS screening.

The MeOH and DCM extracts usually extract a higher proportion of polar and nonpolar compounds, respectively [21, 22]. In quantitative analysis of phytocompounds, LC–MS and GC–MS techniques are utilized to detect nonvolatile and volatile phytocompounds, respectively. The two techniques usually separate compounds using a separation column and then quantify them using mass spectrum according to their mass-to-charge ratio [32, 33]. The LC–MS and GC–MS analyses were, therefore, utilized to quantify phytocompounds that were extracted using MeOH and DCM, respectively.

The LC–MS analysis of the extracts revealed various secondary plant metabolites that possess anti-inflammatory effects. These secondary metabolites included terpenoids, flavonoids, alkaloids, phenolic acids, and phytosterols [34]. The classes of phytochemicals that were detected in this study included flavonoids, phenolic acids, tannins, fatty acids, and phytosterols. Flavonoids are polyphenols that possess a benzopyrone ring with polyphenolic or phenolic groups at distinct positions [35]. Flavonoids can be categorized into multiple subclasses such as flavonols, flavones, flavanones, flavans, isoflavones, neoflavonoids, flavonolignans, flavonoid glycosides, anthocyanidins, and chalcones [36]. The structure of flavonoids comprises C_6_C_3_C_6_ with a heterocyclic oxygenated benzopyran ring and two aromatic rings [37]. There are several health benefits associated with flavonoids, including antidiabetic, antiviral, anti-inflammatory, anticancer, and antioxidant activities [36, 38].

Several mechanisms are involved in the production of anti-inflammatory effects by flavonoids, including enhanced immune mechanisms and ROS scavenging, inhibition of lipoxygenase activity, suppression of leukotriene synthesis, inhibition of synthesis of TNF-α, IL-1β, and IL-6, and suppression of activities of COX-2 and PGE2 [36, 37]. The anti-edema activities of flavonoids are ascribed to their structure. For example, position and number of OH groups, ring unsaturation, −C=O groups, glycosides with high lipophilicity, and methylation [37, 39]. The enhanced anti-edema effect of flavonoids may be explained by the increased number of OH groups in the ring that inhibit lipoxygenase activity. The OCH_3_ group also raises the lipophilicity and bioavailability of flavonoids, which in turn increases the suppression of lipoxygenase activity [37].

The flavonoids that were identified in this study included catechin, genistein, rutin, luteolin, biochanin, eriodictyol, quercetin, and kaempferol. Catechins (flavanols) exert their anti-edema effect by regulating the deactivation of inflammation signaling pathways such as MAPKs (mitogen-activated protein kinases) and nuclear factor-kappa B (NF-κB) pathways [40, 41]. According to reports, genistein (isoflavone) possesses anti-edema properties by scavenging ROS and inhibiting TNF-α, IL-1β, and PGE2 [42]. Rutin (flavonol glycoside) exerts its anti-edema effects by upregulating the expression of inhibitor of IκBα (nuclear factor kappa B) and downregulating levels of TNF-α and IL-6 [43].

The strong anti-edema actions of luteolin (flavone) are achieved by inhibiting NF-κB and STAT3 (signal transducer and the activator of transcription-3) signaling pathways [44]. By modifying the MAPKs and NF-κB signaling pathways, biochanin (isoflavone) prevents the generation of inflammatory cytokines [45]. Eriodictyol (flavanones) possesses anti-inflammatory properties through suppressing the MAPKs and NF-κB signaling pathways [46]. It has been shown that quercetin (flavonol) reduces inflammation by suppressing the NF-κB and MAPK pathways [47], including downregulating expression of IL-6, TNF-α, and IL-1β [48]. Kaempferol, also known as flavonol, inhibits the NF-κB pathway and reduces the levels of TNF-α and IL-1β expression [49].

The phenolic acids were also identified in this study. Phenolic acids are polyphenols that have methoxyl groups, one or more hydroxyl groups, and a carboxylic acid group [50]. The two primary classes of phenolic acids are hydroxycinnamic and hydroxybenzoic acids. Chlorogenic, p-coumaric, and ferulic acids are examples of hydroxycinnamic acids; gallic, syringic, salicylic, protocatechuic, vanillic, ellagic, and gentisic acids are examples of hydroxybenzoic acids [50, 51]. Naturally occurring antioxidants, phenolic acids have a number of medicinal benefits, such as hepatoprotective, anti-allergic, anti-cancer, antibacterial, and antiviral properties. They also have anti-edema properties [52].

Using LC–MS analysis, phenolic acids, including p-coumaric, ferulic, vanillic, and chlorogenic acids, were found in the four extracts. Chlorogenic acid exerts its anti-edema effects by suppressing levels of IL-1β, IL-6, TNF-α, PGE2, and NF‐κB [53, 54]. Ferulic acid reduces inflammation by inhibiting MAPKs and NF-κB signaling pathways, as well as by downregulating levels of IL-1β, TNF-α, and IL-6 [55]. *p*-Coumaric acid inhibits TNF-α and IL-6 to induce anti-edema action [56]. Vanillic acid inhibits the NF-κB pathway and downregulates expression of IL-1β and TNF-α [57, 58].

The LC–MS analysis also identified phytosterols. The steroid skeleton that makes up phytosterols is defined by a saturated bond between C-5 and C-6 sterol moieties. Numerous studies have documented the impressive pharmacological properties of phytosterols, which include antioxidant, chemopreventive, anti-edema, antidiabetic, and antiatherosclerotic activities [59]. This study identified phytosterols such as β-sitosterol and stigmasterol. Beta-Sitosterol exerts its anti-inflammatory effect via scavenging of ROS and inhibition of TNF-α, IL-1β, and IL-6 [60]. Stigmasterol has shown its anti-edema effects by lowering TNF-α levels and blocking activity of the COX-2 enzyme [61].

Fatty acids were also identified using LC–MS analysis. Carboxylic acids that have lengthy aliphatic chains and can be either unsaturated or saturated are known as fatty acids. They are characterized by a methyl group (−CH3) and a carboxyl group (−COOH) [62]. Oleic acid, a monounsaturated omega-9 fatty acid, was identified using LC–MS in this study. Studies have shown that oleic acid reduces edema by suppressing COX-2 activity and inhibiting the NF-κB and MAPK pathways [63].

The GC–MS analysis also detected compounds that have been associated with anti-inflammatory effects, including terpenoids, terpenes, alkaloids, fatty acids, vitamins, phytosterol, and fatty acid methyl ester. Terpenoids are a modified class of terpenes that have distinct functional groups and methyl groups that have undergone oxidation at various positions. Terpenoids are hydrocarbons containing oxygen [64]. Based on their carbon units, terpenes, also known as terpenoids, are categorized as follows: hemiterpenes, monoterpenes, sesquiterpenes, diterpenes, sesterterpenes, triterpenes, and carotenoids/tetraterpenes with 5, 10, 15, 20, 25, 30, and 40 carbon atoms, respectively [65].

The terpenes that were identified in this study included p-cymene (monoterpene), benzene, 2,4-dimethyl-1-(1-methylethyl)- (monoterpene), pseudo phytol < 6E, 10Z -> (diterpene), and olean-18-en-28-oic acid, 3-oxo-, methyl ester (triterpene), while terpenoids included phytol acetate < E -> (diterpenoid), cyperotundone (sesquiterpenoids), and zierone (sesquiterpenoid). Numerous studies have demonstrated that naturally occurring terpenoids and their derivatives have anti-inflammatory properties. Terpenes and terpenoids have been found to downregulate the levels of TNF-α [66].

Alkaloids were also detected using the GC–MS analysis. Alkaloids are naturally occurring organic compounds with nitrogen atoms [67]. Morphinan, 7,8-didehydro-3-methoxy-17-methyl-6, was an alkaloid that was identified in this study. Alkaloids suppress production of histamine, as well as expression of COX-2, IL-1β, and TNF-α, to achieve their anti-inflammatory actions [68, 69].

Fatty acids were also detected using GC–MS analysis. They included methyl p-tert-butyl phenyl acetate and isopropyl hexadecanoate. Fatty acids are documented to possess an anti-edema effect [70]. Using GC–MS analysis, fatty acid methyl esters were also detected in this investigation. These are esters of fatty acids. They are derived through the transesterification of fats with MeOH. It has been documented that they suppress the expression of TNF-α, IL-6, and NF-κB [3, 71]. Methyl palmitate and olean-18-en-28-oic acid, 3-oxo-, methyl ester are some of the fatty acid methyl esters that were identified in this study.

Vitamins were also detected using GC–MS analysis in this study. Vitamins exert their anti-edema effects through the scavenging of ROS [72]. Vitamin E and beta and gamma tocopherol were some of the vitamins that were identified in this study. Other phytocompounds that were detected using GC–MS were phytosterols. As was earlier discussed, phytosterols have been demonstrated to possess anti-edema properties. Gamma-sitosterol is one of the phytosterols that was identified using GC–MS in this study.

According to this investigation, at the higher dosage of 200 mg/kg bw, the MeOH stem bark extract exhibited superior activity in comparison to the other three extracts. In general, it was evident that the MeOH extracts were more effective as compared with the DCM extracts. The better anti-edema effect of MeOH stem bark extract could be attributed to the polar phytocompounds. It is also possible that phytocompounds that had better anti-inflammatory effects were highly concentrated in the stem bark.

## 5. Conclusions

The DCM and MeOH leaf and stem bark extracts of *F*. *sycomorus* at a dose level of 200 mg/kg bw have appreciable anti-inflammatory activities in mice. The extracts contained phytocompounds associated with anti-inflammatory activities; hence, they are potential alternative anti-inflammatory agents. However, histological and safety profiles of the studied extracts should be evaluated, and the anti-inflammatory activities of isolated extract fractions assessed.

## Figures and Tables

**Table 1:** Protocol for anti-inflammatory assay for DCM/MeOH leaf and stem bark extracts.

Status	Treatment
Group I	Carrageenan + normal saline + 5% DMSO
Group II	Normal saline + 5% DMSO
Group III	Carrageenan + diclofenac (15 mg/kg bw)
DCM/MeOH extract low dose	Carrageenan + *F. sycomorus* extract (50 mg/kg bw)
DCM/MeOH extract medium dose	Carrageenan + *F. sycomorus* extract (100 mg/kg bw)
DCM/MeOH extract maximal dose	Carrageenan + *F. sycomorus* extract (200 mg/kg bw)

**Table 2:** In vivo anti-inflammatory activity of DCM stem bark extract of *F. sycomorus*.

Group	% change in paw diameter			
	1 h	2 h	3 h	4 h
Normal control	0.00 ± 0.00^d^ (0.00)	0.00 ± 0.00^c^ (0.00)	0.00 ± 0.00^e^ (0.00)	0.00 ± 0.00^e^ (0.00)
Negative control	101.76 ± 0.39^a^ (−1.76)	103.70 ± 0.42^a^ (−3.70)	106.00 ± 0.16^a^ (−6.00)	107.15 ± 0.15^a^ (−7.15)
Positive control	95.17 ± 0.42^b^ (4.83)	92.09 ± 0.20^b^ (7.91)	89.23 ± 0.17^bc^ (10.77)	86.37 ± 0.45^bc^ (13.63)
50 mg/kg bw	95.98 ± 0.39^b^ (4.02)	92.41 ± 0.55^b^ (7.59)	89.64 ± 0.23^b^ (10.36)	87.52 ± 0.42^b^ (12.48)
100 mg/kg bw	93.49 ± 0.46^c^ (6.51)	91.09 ± 0.40^b^ (8.91)	88.26 ± 0.52^cd^ (11.74)	86.31 ± 0.49^bc^ (13.69)
200 mg/kg bw	93.32 ± 0.35^c^ (6.68)	91.31 ± 0.43^b^ (8.69)	87.75 ± 0.34^d^ (12.25)	85.53 ± 0.28^c^ (14.47)

*Note:* Descriptive statistics are represented as mean plus or minus standard error of the mean. The percentage of inflammation inhibition is presented by the values in parentheses. There is no statistically significant variation between descriptive statistics with the same letter along the column, using one-way ANOVA and Tukey's multiple comparisons (*p* > 0.05).

**Table 3:** In vivo anti-inflammatory activity of MeOH stem bark extract of *F. sycomorus*.

Treatment	% change in paw diameter			
	1 h	2 h	3 h	4 h
Normal control	0.00 ± 0.00^d^ (0.00)	0.00 ± 0.00^e^ (0.00)	0.00 ± 0.00^e^ (0.00)	0.00 ± 0.00^e^ (0.00)
Negative control	101.35 ± 0.38^a^ (−1.35)	103.49 ± 0.40^a^ (−3.49)	104.46 ± 0.43^a^ (−4.46)	106.59 ± 0.27^a^ (−6.59)
Positive control	94.00 ± 0.43^b^ (6.00)	91.43 ± 0.36^b^ (8.57)	88.44 ± 0.40^b^ (11.56)	86.73 ± 0.21^b^ (13.27)
50 mg/kg bw	94.88 ± 0.17^b^ (5.12)	91.54 ± 0.33^b^ (8.46)	88.99 ± 0.26^b^ (11.01)	87.21 ± 0.22^b^ (12.79)
100 mg/kg bw	94.07 ± 0.25^b^ (5.93)	89.20 ± 0.15^c^ (10.80)	87.08 ± 0.15^c^ (12.92)	85.39 ± 0.21^c^ (14.61)
200 mg/kg bw	90.33 ± 0.18^c^ (9.67)	87.32 ± 0.19^d^ (12.68)	85.17 ± 0.17^d^ (14.83)	83.46 ± 0.26^d^ (16.54)

*Note:* Descriptive statistics are represented as mean plus or minus standard error of the mean. The percentage of inflammation inhibition is presented by the values in parentheses. There is no statistically significant variation between descriptive statistics with the same letter along the column, using one-way ANOVA and Tukey's multiple comparisons (*p* > 0.05).

**Table 4:** In vivo anti-inflammatory activity of DCM leaf extract of *F. sycomorus*.

Group	Percentage change in paw diameter			
	1 h	2 h	3 h	4 h
Normal control	0.00 ± 0.00^c^ (0.00)	0.00 ± 0.00^c^ (0.00)	0.00 ± 0.00^d^ (0.00)	0.00 ± 0.00^e^ (0.00)
Negative control	101.98 ± 0.64^a^ (−1.98)	103.72 ± 0.22^a^ (−3.72)	104.50 ± 0.25^a^ (−4.50)	106.44 ± 0.37^a^ (−6.44)
Positive control	94.71 ± 0.35^b^ (5.29)	91.76 ± 0.37^b^ (8.24)	88.36 ± 0.40^c^ (11.64)	87.09 ± 0.27^cd^ (12.91)
50 mg/kg bw	95.50 ± 0.25^b^ (4.50)	92.37 ± 0.46^b^ (7.63)	90.80 ± 0.42^b^ (9.20)	89.05 ± 0.41^b^ (10.95)
100 mg/kg bw	95.63 ± 0.28^b^ (4.37)	91.85 ± 0.46^b^ (8.15)	90.07 ± 0.14^b^ (9.93)	87.71 ± 0.25^c^ (12.29)
200 mg/kg bw	95.28 ± 0.28^b^ (4.72)	91.81 ± 0.26^b^ (8.19)	88.52 ± 0.19^c^ (11.48)	86.27 ± 0.23^d^ (13.73)

*Note:* Descriptive statistics are represented as mean plus or minus standard error of the mean. The percentage of inflammation inhibition is presented by the values in parentheses. There is no statistically significant variation between descriptive statistics with the same letter along the column, using one-way ANOVA and Tukey's multiple comparisons (*p* > 0.05).

**Table 5:** In vivo anti-inflammatory activity of methanol leaf extract of *F*. *sycomorus*.

Group	Percentage change in paw diameter			
	1 h	2 h	3 h	4 h
Normal control	0.00 ± 0.00^c^ (0.00)	0.00 ± 0.00^e^ (0.00)	0.00 ± 0.00^d^ (0.00)	0.00 ± 0.00^f^ (0.00)
Negative control	101.57 ± 0.25^a^ (−1.57)	103.52 ± 0.25^a^ (−3.52)	104.51 ± 0.54^a^ (−4.51)	106.64 ± 0.19^a^ (−6.64)
Positive control	96.09 ± 0.23^b^ (3.91)	92.59 ± 0.46^d^ (7.41)	89.30 ± 0.54^c^ (10.70)	86.86 ± 0.17^d^ (13.14)
50 mg/kg bw	97.33 ± 0.38^b^ (2.67)	94.48 ± 0.22^b^ (5.52)	91.25 ± 0.18^b^ (8.75)	88.97 ± 0.26^b^ (11.03)
100 mg/kg bw	96.58 ± 0.41^b^ (3.42)	93.77 ± 0.33^bc^ (6.23)	89.55 ± 0.30^c^ (10.45)	87.93 ± 0.24^c^ (12.07)
200 mg/kg bw	96.06 ± 0.32^b^ (3.94)	93.11 ± 0.08^cd^ (6.89)	88.17 ± 0.42^c^ (11.83)	85.23 ± 0.34^e^ (14.77)

*Note:* Descriptive statistics are represented as mean plus or minus standard error of the mean. The percentage of inflammation inhibition is presented by the values in parentheses. There is no statistically significant variation between descriptive statistics with the same letter along the column, using one-way analysis of variance and Tukey's multiple comparisons (*p* > 0.05).

**Table 6:** LC–MS analysis of MeOH stem bark extract of *F*. *sycomorus*.

RT (min)	Compound name	Molecular formula	Chemical class	Relative abundance (%)
1.31	Ferulic	C_10_H_10_O_4_	Phenolic acid	10.5
1.46	Catechin	C_15_H_14_O_6_	Tannins	16.87
1.59	Chlorogenic acid	C_16_H_18_O_9_	Ester	14.02
1.64	Oleic acid	C_18_H_34_O_2_	Fatty acid	8.38
1.69	Vanillic acid	C_8_H_8_O_4_	Phenols	7.77
2.95	*p*-Coumaric	C_9_H_8_O_3_	Phenols	0.29
3.28	Procyanidin A1	C_30_H_24_O_12_	Flavonols	0.51
4.88	Procyanidin B2	C_30_H_26_O_12_	Flavonols	1.78
5.08	Rutin	C_27_H_30_O_16_	Phenolic	0.29
6.07	Arachidic acid	C_20_H_40_O_2_	Fatty acid	2.82
8.37	Genistein	C_15_H_10_O_5_	Isoflavones	11.32
8.88	Eriodictyol	C_15_H_10_O_6_	Flavonoid	8.66
8.99	Kaempferol	C_15_H_10_O_6_	Flavonoid	4.47
9.04	Biochanin A	C_16_H_12_O_5_	Flavonoid	2.68
9.13	Luteolin	C_15_H_10_O_6_	Flavonoid	0.63
9.21	Apigenin	C_15_H_10_O_5_	Flavonoid	0.45
9.62	Quercetin	C_15_H_10_O_7_	Flavonoid	4.8
10.77	Campesterol	C_28_H_48_O	Sterol	0.35
10.83	Stigmasterol	C_29_H_48_O	Phytosterol	0.09
10.94	Beta-Sitosterol	C_29_H_50_O	Sterol	3.3

Abbreviation: RT = retention time.

**Table 7:** LC–MS analysis of MeOH leaf extracts of *F. sycomorus*.

RT (min)	Compound name	Molecular formula	Chemical class	Relative abundance (%)
1.32	Ferulic acid	C_10_H_10_O_4_	Phenolic	14.27
1.46	Catechin	C_15_H_14_O_6_	Flavonoids	8.67
1.51	4-Hydroxycinnamic acid	C_9_H_8_O_3_	Phenols	4.65
1.54	Oleic acid	C_18_H_34_O_2_	Fatty acid	2.74
1.58	Chlorogenic acid	C_16_H_18_O_9_	Ester	5.24
1.62	Oleic acid	C_18_H_34_O_2_	Fatty acid	1.97
4.60	Procyanidin A1	C_30_H_24_O_12_	Flavonols	3.89
4.77	Procyanidin B2	C_30_H_26_O_12_	Flavonols	1.86
4.87	Rutin	C_27_H_30_O_16_	Phenolic	7.2
8.45	Genistein	C_15_H_10_O_5_	Isoflavones	10.69
8.90	Eriodictyol	C_15_H_12_O_6_	Flavonoids	4.17
8.94	Kaempferol	C_15_H_10_O_6_	Flavonoids	5.62
9.00	Biochanin A	C_16_H_12_O_5_	Flavonoids	7.85
9.06	Luteolin	C_15_H_10_O_6_	Flavonoids	5.91
9.20	Apigenin	C_15_H_10_O_5_	Flavonoids	2.59
9.57	Quercetin	C_15_H_10_O_7_	Flavonoids	5.31
10.77	Campesterol	C_28_H_48_O	Sterol	4.87
10.93	Beta-Sitosterol	C_29_H_50_O	Sterol	2.49

Abbreviation: RT = retention time.

**Table 8:** GC–MS analysis of DCM stem bark extract of *F*. *sycomorus*.

RT (min)	Compound name	Molecular formula	Chemical class	Relative abundance (%)
6.69	2,5-Dihydrofuran	C_4_H_6_O	Furan	0.01
9.47	3,6,6-Trimethyl-cyclohex-2-enol	C_9_H_16_O	Alcohol	0.15
9.69	Pyrrolidine	C_4_H_9_N	Cyclic amine	0.01
11.15	Morphinan,7,8-didehydro-3-methoxy-	C_19_H_23_NO	Alkaloid	0.01
13.50	*p*-Cymene	C_10_H_14_	Monoterpene	0.13
13.79	*1*-Phenyl-1-butene	C_10_H_12_	Alkene	0.01
14.03	Cyclopentene < 3,5-dimethylene-1	C_10_H_14_	Alkene	0.12
14.75	Benzene,2,4-dimethyl-1-(	C_11_H_16_	Monoterpene	0.10
16.01	6-Methyl-4-indanol	C_10_H_12_O	Alcohol	0.01
23.51	Methyl palmitate	C_17_H_34_O_2_	Fatty acid	0.08
24.38	Isopropyl palmitate	C_19_H_38_O_2_	Fatty acid	0.08
26.49	3,6-Dimethylpiperazine-2,5-dione	C_6_H_10_N_2_O_2_	Pyrazine	0.06
27.90	E-8-Methyl-9-tetradecen-1-ol acetate	C_17_H_32_O_2_	Alcohol	0.15
31.80	1-Methyl-2-phenylbenzimidazole	C_14_H_12_N_2_	Benzimidazole	0.25
31.12	2,3,6-Trimethylhept-5-en-1-ol	C_10_H_20_O	Alcohol	0.36
38.98	Beta-Sitosterol	C_29_H_50_O	Phytosterol	0.17
41.31	13,27-Cyclours-11-en-3-ol, acetate	C_32_H_50_O_2_	Alcohol	0.35
42.19	Zierone	C_15_H_22_O	Sesquiterpenoid	14.93
43.58	A-Neogammacer-22(29)-en-3-ol	C_32_H_52_O_2_	Alcohol	82.60
46.22	Olean-18-en-28-oic acid, 3-oxo-, methyl ester	C_31_H_48_O_3_	Fatty acid methyl ester	0.42

Abbreviation: RT = retention time.

**Table 9:** GC–MS analysis of DCM leaf extract of *F. sycomorus*.

RT (min)	Compound name	Molecular formula	Chemical class	Relative abundance (%)
6.71	2-Hexene,2,5,5-trimethyl	C_9_H_18_	Alkenes	0.13
7.47	Oxalic acid	C_14_H_22_O_4_	Carboxylic acid	0.34
9.47	2-Butenal,3-methyl-	C_5_H_8_O	Aldehyde	1.85
13.74	*p*-Cymene	C_10_H_14_	Monoterpene	0.33
18.92	Methyl p-tert-butyl phenyl acetate	C_13_H_18_O_2_	Fatty acid	0.73
22.52	Phytol	C_22_H_42_O_2_	Diterpenoid	1.27
25.17	Acetamide,2-cyano-	C_3_H_4_N_2_O	Esters	0.37
26.67	Benzaldehyde,2-nitro-,diaminomethylidenhydrazone	C_8_H_9_N_5_O_2_	Aldehyde	0.52
25.39	2,2,3,4-Tetramethyl-3-ol,isobutylpentyl ether	C_9_H_20_O	Fatty alcohol	0.55
22.79	Citronellyl valerate	C_15_H_28_O_2_	Esters	0.65
23.51	Methyl palmitate	C_17_H_34_O_2_	Fatty acid	0.77
24.05	Octadecagon < n->	C_18_H_38_O	Fatty alcohol	0.76
31.12	Pseudo phytol < 6E, 10Z->	C_20_H_36_O	Diterpene	10.15
39.52	Benzo h quinolone,2,4-dimethyl-	C_15_H_13_N	Aldehyde	0.93
33.54	Z-11(13-Methyl) tetradecen-1-ol acetate	C_17_H_32_O_2_	Hydrocarbon	3.75
35.15	Vitamin E	C_29_H_50_O	Vitamin E	6.61
37.32	Beta and gamma tocopherol	C_28_H_48_O_2_	Vitamin E	1.32
38.98	Gamma-Sitosterol	C_29_H_50_O_2_	Phytosterol	3.72
39.14	4,4,6a,6b,8a,11,11,14b-Octamethyl-	C_30_H_48_O	Triterpenes	3.17
39.72	Cyperotundone	C_15_H_22_O	Sesquiterpene	5.97
40.19	13,27-Cyclours-11-en-3-ol, acetate	C_32_H_50_O_2_	Alcohol	6.17
40.78	18, 19-Secolupan-3-ol, (3. beta. 17)	C_30_H_54_O	Alcohol	2.48
42.12	Zierone	C_15_H_22_O	Sesquiterpene	40.68
45.86	Olean-18-en-28-oic acid, 3-oxo-, methyl ester	C_31_H_48_O_3_	Triterpenes	6.72
49.58	2-Ethylacridine	C_15_H_13_N	Fatty acid	0.06

Abbreviation: RT = retention time.

## Data Availability

The data generated and/or analyzed during the current study are available from the corresponding author on reasonable request.
